# Exploring the Surveillance Potential of Mortality Data: Nine Years of Bovine Fallen Stock Data Collected in Catalonia (Spain)

**DOI:** 10.1371/journal.pone.0122547

**Published:** 2015-04-15

**Authors:** Anna Alba, Fernanda C. Dórea, Lucas Arinero, Javier Sanchez, Ruben Cordón, Pere Puig, Crawford W. Revie

**Affiliations:** 1 Centre de Recerca en Sanitat Animal (CReSA)—Institut de Recerca i Tecnologia Agroalimentàries (IRTA), Campus UAB, 08193 Bellaterra, Barcelona, Spain; 2 Department of Disease Control and Epidemiology, National Veterinary Institute (SVA), Uppsala, Sweden; 3 Ministry of Agriculture, Livestock, Fisheries, Food and Natural Environment, Government of Catalonia, Barcelona, Spain; 4 Centre for Veterinary Epidemiological Research, AVC, University Prince Edward Island (UPEI), Charlottetown, Canada; 5 Departament de Matemàtiques, Universitat Autònoma de Barcelona, Cerdanyola del Vallès, Barcelona, Spain; Auburn University, UNITED STATES

## Abstract

The potential of fallen stock data to monitor the health status of animal populations has been noted in previous studies. However, further research is required to implement these systems for surveillance. This work presents a novel approach to determining the baselines associated with bovine fallen stock, comparing patterns between subpopulations and identifying subpopulations in which an abnormal event may occur. This study was based on data from 193,873 disposal visits carried out between 2004 and 2012 across a total of 2,991 bovine farms. Proxy measurements such as the number of collections carried out and the weight of carcasses collected were used. Both outcomes were aggregated weekly at different geographical scales for three production types (beef cattle, dairy cattle and heifer fattening). The analysis of these data combined autoregressive integrated moving average modelling and hierarchical time series methods.The three production types exhibited historical baselines that differed notably from one another. Based on the 757 beef cattle farms monitored, the mean number of collections registered per week at the regional level was 37 (range: 10–83). This series was relatively constant over time and showed a marked yearly seasonality. In contrast, for the 426 dairy cattle farms the mean number of disposal visits registered weekly was 121 (range: 71–180), showing half-yearly and yearly seasonality and a marked increase over the period monitored. From the 1,808 heifer fattening farms the mean number of disposal visits was 248 (range: 166–357) and the pattern presented a marked alternating trend over time. These patterns were assessed and compared at regional, provincial, county and municipal levels. The use of hierarchical time series approaches appeared to be a useful tool for comparing the patterns within different subpopulations over time as well as for assessing the spatial extent to which various abnormal events could be detected.

## Introduction

The recent growth in the field of veterinary syndromic surveillance has led to the exploration of a range of data sources, some of which do not precede diagnoses (such as abattoir condemnation and mortality data) [1–8], but which have been previously under-utilized for continuous animal health monitoring. The use of existing digital animal health data to obtain information in near real time is now possible, as a consequence of developments in the areas of data mining, machine learning and statistical analysis [9].

Data related to fallen stock constitute one of these potential sources, still unexplored in many places, which could signal a broad range of animal health problems and can be easily monitored [4–7]. In the European Union, the collection of fallen stock at farm level has been compulsory since 2002 [10, 11]. Fallen stock include animals that have died as a result of many health or nutritional problems, natural disasters or that have been killed on a farm for reasons other than for human consumption. In some regions, such as Catalonia (in North-Eastern Spain), these data are automatically registered by animal health authorities and carcass disposal services. Although the unspecific nature of these data makes it impossible to group abnormal events into syndromes, the use of mortality data provides broad coverage in the target population, is readily computerized, and continuously updated. However, before attempting to use such data for the detection of abnormal events in real time, it is essential to determine the best parameters and level of aggregation that should be adopted in any analyses. Studying the basal patterns from available historical data can help in determining those algorithms that can provide accurate forecasts for the normal pattern of events that might be expected in a given target population [2, 5, 8].

The objective of this work is to assess the nature of the data collected on farm mortality in Catalonia over a nine-year period, in any attempt to provide new approaches to build the initial components of a syndromic system based on farm-mortality data. This study analyses the time series patterns associated with bovine fallen stock registered between 2004 and 2012 at both regional and various subpopulation levels. The analyses combine classical approaches, such as autoregressive integrated moving average (ARIMA) modelling with previous adjustment for seasonal and trend components, with novel approach, the plotting of hierarchical time series. This work proposes an approach for determining robust algorithms that can extract the basal patterns of bovine fallen stock at large scale, can simply compare the baselines of different subpopulations and visually identify at finer scales those subpopulations in which abnormal events are likely to occur.

## Material and Methods

### Population of study

The total cattle population of Catalonia destined for production or reproduction is composed of 6,952 farms with a census of 634,412 bovine animals (official data registered in 2012, DAAM). The cattle population in this region is heterogeneous in relation to the sizes of farm, husbandry systems and environmental conditions.

**Fig 1:**
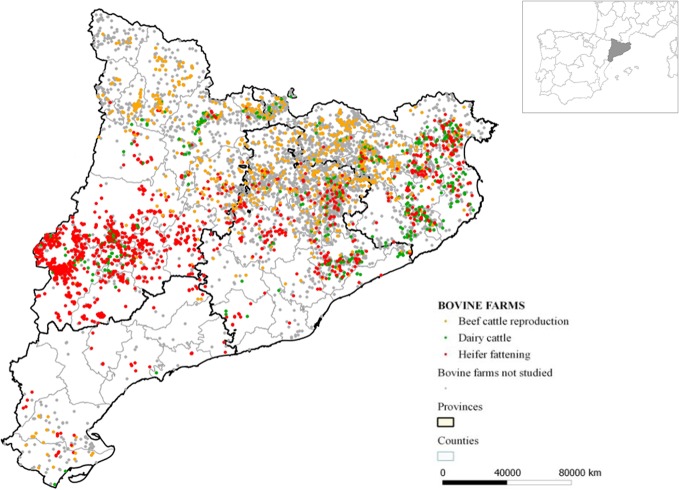
Spatial distribution of the cattle populations included in this study.

This study was performed using retrospective data associated with bovine fallen stock, collected from a total of 2,991 farms in Catalonia between 2004 and 2012; representing approximately 43% of the cattle farms in the region. The data gathered came from 757 (25%) beef cattle reproduction farms, 426 (14%) dairy cattle farms, and 1,808 (61%) heifer fattening farms. These data were collected in 531 municipalities belonging to 37 counties from 4 provinces in Catalonia (see Fig 1).

### Data sets and sources used

To study the patterns of bovine fallen stock, two different databases were merged and analyzed: (1) a dataset of fallen stock registered daily by the carcass disposal services that operate in this region; and (2) the official data of cattle populations registered yearly by the official services.

These two datasets were merged using the unique farm identifier as a primary key. The data are centralized weekly at a regional level by the animal health authorities of the Department of Agriculture, Livestock, Fishery, Nutrition and Natural Environment of Catalonia. The datasets were depurated and merged to obtain a unique final database. The final dataset included the following attributes for each fallen stock case: a unique identifier for each bovine farm, its exact location (i.e. coordinates, municipality, county and province), the farm capacity (e.g. maximum number of bovines that the farm can raise) and type of production, as given in the annual census for each farm. In addition, for each carcass collection, the date of the disposal collection and the weight (in kg) of the carcass(es) collected on that date was recorded.

### Exploratory descriptive analysis

Before attempting to model the data, a descriptive analysis was conducted. This aimed to filter and identify appropriate outcome variables to describe the fallen stock patterns and extract their overall statistics (mean, median, range, variance and autocovariance function to assess the covariance of these processes at pairs of time points).

### Time series study

At the time that the study was conducted the exact number of bovine carcasses collected by farm was not available. Consequently, to provide better insights related to mortality patterns, two types of observations were examined in parallel as outcome variables: (i) the number of visits carried out by the carcass disposal services (V), and (ii) the number of kilograms of bovine carcasses collected (kg). Both sets of observations were aggregated by week at different geographical scales for the three production types of cattle farms mentioned above. The aggregation at week level was considered adequate to provide information in the event of detecting an abnormal peak and avoid the distortion of fallen stock pattern that could exist at the daily level due to the lack of carcasses collections carried out over the weekend.

The analyses of these time series of V and kg were conducted in three phases: (a) identification of ARIMA models with a previous adjustment for seasonal component from data registered between 2004 and 2011 at regional level, forecasts for 2012 and model tests against the real observations registered during that year, (b) creation and plotting of hierarchical time series structures, with the aim of visualizing patterns at the four geographical levels and comparing different subpopulations, and (c) interpretation of the time series patterns analysed.

### Identification of ARIMA models at regional level and forecasting

Initially the historical data to fit the models were divided in two parts. The data collected between 2004 and 2011 were used as training data to identify the ARIMA models, and the data of 2012 were used to test the models identified. The baselines of both outcome variables (V and kg) were studied at the regional level for the main production types using ARIMA modelling with an adjustment for any observed seasonality and/or trend. This method allows for the representation of a wide range of data and can be used to assess a large number of parameters and combination of terms. ARIMA modelling, based on normally distributed patterns, has been demonstrated to be a robust approach to fit models for those count time series that show regular patterns and when most of the values are greater than 10 [11–13].

The process of fitting each series to an appropriate model consisted of: (a) the study and adjustment of its possible seasonal patterns and/or trends, (b) the identification of any autoregressive/moving average processes involved with their respective order, and finally (c) the diagnostic checking of standarized residuals in the model proposed.

Initially, to detect the existence of possible seasonal patterns or trends, time series plots of the weekly observations aggregated at regional level were explored. If seasonal and/or trend components were detected, they were defined and subtracted from the original series. The adjustment of these components facilitated the identification of more parsimonious ARIMA models. Mathematically, the overall set of observations was expressed as X_t_ as follows (1):

$${\text{X}}_{\text{t}}=\mu +\alpha \;\text{cos}\left(\omega t\right)+\beta \;\text{sin}\left(\omega t\right)+\ldots +\delta \left(\text{t}\right)+{\text{Y}}_{\text{t}}$$ 1

X_t_ contained the following terms: the trigonometric covariates correspond to the possible seasonal components (defined as trigonometric covariates) in which the frequency ω was expressed as ω = 2π/T and T was the value of the seasonal periodicity. Eventually, if several seasonal or cyclical patterns were observed, several trigonometric components were added in accordance with the series; δ(t) corresponding to the eventual trend component initially modelled as a linear pattern; and Y_t_ representing the remaining ARIMA model. The coefficients of seasonality and trends were estimated and tested by the method of least squares using a multiple linear regression model.

Subsequently, using correlograms of these series without seasonal variation and also assessing the plausibility of different models proposed by the automatic system, the remaining ARIMA(p,d,q) model was determined. This remaining model Y_t_ was expressed as (2):

$${\text{Y}}_{\text{t}}=\varphi_{0}+\varphi_{1}{\text{Y}}_{\text{t}-1}+\ldots +\varphi_{\text{p}}{\text{Y}}_{\text{t}-\text{p}}+\ldots +{\text{Z}}_{\text{t}}+\theta_{1}{\text{Z}}_{\text{t}-1}+\ldots +\theta_{\text{q}}{\text{Z}}_{\text{t}-\text{q}}$$ 2

Where p was the order of the autoregressive part of the model and q indicated the order of the moving average part.

To identify the best order of the different components of the ARIMA model in each case, the principle of parsimony and the corrected Akaike Information Criteria (AICc) were taken into account. Subsequently, diagnostic checking was conducted, using standarized residuals with their correspondent autocorrelation and partial autocorrelation.

Subsequently, using the models determined for each production type at regional level, one-step-ahead forecasts with their corresponding 95% confidence interval range were calculated for the next 52 weeks of 2012 and contrasted with the actual observations.

The base package of the software R [14] with *R Studio* as an integrated development environment [15] was used for the analyses.

### Hierarchical time series

Complementary studies involving hierarchical time series (HTS) plots were performed to visually explore the time series patterns at finer spatial scales. These studies were based on a method proposed by Hyndman et al. [15–18] in relation to HTS. HTS structures were fitted with the outcome variables V and kg for each production type at the four geographical levels. This method of plotting aims not only to provide a fast method by which to compare baseline patterns for different subpopulations over time, but also facilitates the identification of the spatial extent of irregular patterns previously detected at regional level.

The design of HTS structures combines two matrices that contain two pieces of information: observations at the bottom-level of the time series (in this case: municipalities), and the hierarchical organization which dictates how the observations of the municipalities series were aggregated within the county, province and ultimately the regional levels. The notation of this hierarchy is written as (3):

$$Z_{t}=Sz_{k,t}$$ 3

Where *Z*_*t*_ is a vector of all the observations in the hierarchy at time t, *S* is the summing matrix, and *z*_*k*,*t*_ is a vector of all observations in the bottom level of the hierarchy at time t.

The ‘hts’ package within R supports the creation of these HTS and provides a means to plot these structures [16–17].

### Initial interpretation regarding the main time series patterns generated

Finally, to obtain plausible explanations of the overall patterns identified and support their use in forecasting, some initial inferences were made on the basis of available scientific knowledge. Moreover, to gain a better understanding of the different contexts associated with each of the subpopulations studied, this knowledge was complemented by carrying out interviews with veterinary practitioners and technical staff involved directly in the data collection.

## Results

### Exploratory data analysis

Between 2004 and 2012 the analyzed data included a total of 193,873 disposal visits carried out at 2,991 bovine farms with a total of 42.4 million kilograms of carcasses collected. In beef cattle reproduction were carried out 17,620 visits (~9%), in dairy cattle farms 60,208 visits (~31%), and in heifer fattening farms 119,535 visits (~62%), collecting a total of 4.9, 17.2 and 20.3 million kilograms, respectively. The median number of bovines in these farms was 199 (range:1 to 2,110) for beef cattle reproduction, 241 (1 to 3,436) for dairy cattle, and 336 (2 to 3,000) for heifer fattening.

A summary by province of the key characteristics of data gathered from cattle farms that were included in this study is provided in Table 1.

**Table 1:** Summary of the basic characteristics of the cattle farms included in the study according to their type of production and location by province.

**Basic characteristics**	**Production type**	**Province 1**	**Province 2**	**Province 3**	**Province 4**	**Catalonia**
**Number of farms**	**BCR**	215 (28%)	253 (33%)	268 (35%)	21 (3%)	757 (100%)
	**DA**	94 (22%)	196 (46%)	133 (31%)	3 (1%)	426 (100%)
	**FAH**	249 (14%)	206 (11%)	1310 (72%)	43 (2%)	1808 (100%)
**Total animal capacity**	**BCR**	34130 (30%)	30122 (27%)	44413 (39%)	4527 (4%)	113182 (100%)
	**DA**	14814 (18%)	3449 (4%)	32440 (39%)	473 (1%)	82176 (100%)
	**FAH**	68212 (17%)	48630 (12%)	279358 (68%)	11955 (3%)	408155 (100%)
**Number of visits of carcass collection (2004–2012)**	**BCR**	6078 (35%)	5360 (31%)	5474 (31%)	507 (3%)	17419 (100%)
	**DA**	9975 (17%)	25688 (45%)	21490 (37%)	295 (1%)	57448 (100%)
	**FAH**	18017 (15%)	17533 (15%)	79456 (67%)	4000 (3%)	119006 (100%)
**Millions of Kilograms of carcasses collected (2004–2012)**	**BCR**	1.7 (35%)	1.5 (31%)	1.6 (33%)	0.2 (4%)	4.9 (100%)
	**DA**	2.7 (16%)	7.2 (42%)	7.1 (41%)	0.1 (1%)	17.2 (100%)
	**FAH**	3.5 (17%)	0.2 (1%)	13.3 (66%)	0.7 (3%)	20.3 (100%)

BCR: beef cattle reproduction; DA: dairy cattle; FAH: fattening heifers.

The percentage values (in brackets) were calculated by row (i.e. % in each province versus region)

**Fig 2:**
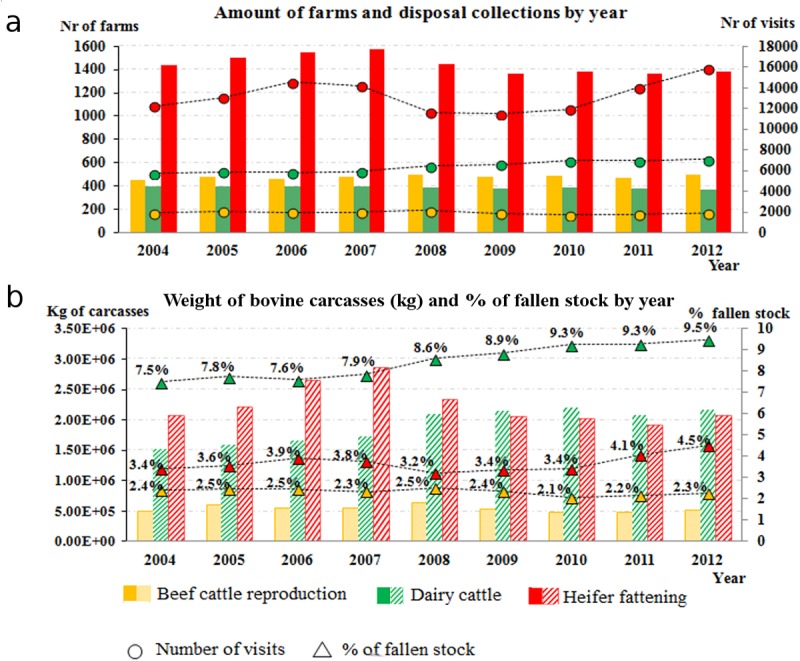
Trend by year of bovine fallen stock for each production type.

The yearly variation in bovine fallen stock according to the number of farms and visits for each production type is illustrated in Fig 2 (above), while the total kilograms of carcasses collected and the percentage of total fallen stock is represented in Fig 2 (below).

In Fig 2 (a) represents the number of farms followed and number of carcass disposal visits carried out; while (b) shows the total kilograms of bovine carcasses collected and the proportion of fallen stock (in %) according to the estimated total bovine capacity.

### Identification of ARIMA models at regional level and forecasting

ARIMA models with trigonometric seasonal and trend component adjusted were defined at regional level for V and kg for the three production types (Table 2).

**Table 2:** ARIMA models with trigonometric seasonal and trend component adjusted defined at regional level for V and kg for each production type.

**Models for weekly number of disposal visits at regional level (V)**								
**Beef cattle reproductionARIMA (1,0,1)**	${\text{X}}_{\text{t}}=37.23+6.30\;\cos\left(\frac{2\pi \text{t}}{52}\right)+13.10\text{sin}\left(\frac{2\pi \text{t}}{52}\right)-2.90\text{cos}\left(\frac{2\pi \text{t}}{26}\right)+{\text{Y}}_{\text{t}}$							
	s.e.	1.17	0.93	0.95	0.64			
	Y_t_ = 0.93Y_t–1_ + Z_t_ − 0.77Z_t–1_							
	s.e.	0.03	0.06					
	AICc = 2836.23							
**Dairy cattle ARIMA (1,0,1)**	${\text{X}}_{\text{t}}=104.68+0.08\text{t}+9.88\;\cos\left(\frac{2\pi \text{t}}{52}\right)-10.70\text{sin}\left(\frac{2\pi \text{t}}{52}\right)+9.80\text{sin}\left(\frac{2\pi \text{t}}{26}\right)+{\text{Y}}_{\text{t}}$							
	s.e.	2.15	0.01	1.29	1.31	1.06		
	Y_t_ = 0.86Y_t–1_ + Z_t_ − 0.75Z_t–1_							
	s.e.	0.06	0.08					
	AICc = 3293.16							
**Fattening heifers ARIMA (0,1,1)**	${\text{X}}_{\text{t}}=13.44\;\cos\left(\frac{2\pi \text{t}}{52}\right)-9.20\text{sin}\left(\frac{2\pi \text{t}}{52}\right)+{\text{Y}}_{\text{t}}$							
	s.e.	2.65	2.69					
	Y_t_ − Y_t–1_ = Z_t_ – 0.81Z_t–1_							
	s.e.	0.03						
	AICc = 3704.60							
**Models for kilograms of carcasses collected by week at regional level (kg)**								
**Beef cattle reproductionARIMA (1,0,1)**	${\text{X}}_{\text{t}}=10591.3+2729.5\;\cos\left(\frac{2\pi \text{t}}{52}\right)+3861.70\text{sin}\left(\frac{2\pi \text{t}}{52}\right)-926.90\;\text{cos}\left(\frac{2\pi \text{t}}{26}\right)+{\text{Y}}_{\text{t}}$							
	s.e.	470.63	372.07	379.60	253.56			
	Y_t_ = 0.93Y_t–1_ + Z_t_ − 0.76Z_t–1_							
	s.e.	0.03	0.05					
	AICc = 7788.34							
**Dairy cattle ARIMA (1,0,1)**	${\text{X}}_{\text{t}}=28391.20+36.50\text{t}+1433.64\;\cos\left(\frac{2\pi \text{t}}{52}\right)-3601.23\text{sin}\left(\frac{2\pi \text{t}}{52}\right)-2691.53\text{sin}\left(\frac{2\pi \text{t}}{26}\right)+{\text{Y}}_{\text{t}}$							
	s.e.	1464.62	6.16	474.39	484.89	402.07		
	Y_t_ = 0.96Y_t–1_ + Z_t_ − 0.89Z_t–1_							
	s.e.	0.03	0.04					
	AICc = 8360.59							
**Fattening heifers ARIMA (0,1,1)**	${\text{X}}_{\text{t}}=4149.8\;\cos\left(\frac{2\pi \text{t}}{52}\right)-1602.56\text{sin}\left(\frac{2\pi \text{t}}{52}\right)+{\text{Y}}_{\text{t}}$							
	s.e.	517.7	527.54					
	Y_t_ – Y_t–1_ = Z_t_ – 0.83Z_t–1_							
	s.e.	0.03						
	AICc = 8161.72							

s.e.: standard errors for the respective coefficient

AICc: Akaike information criterion with a correction for finite sample sizes

**Fig 3:**
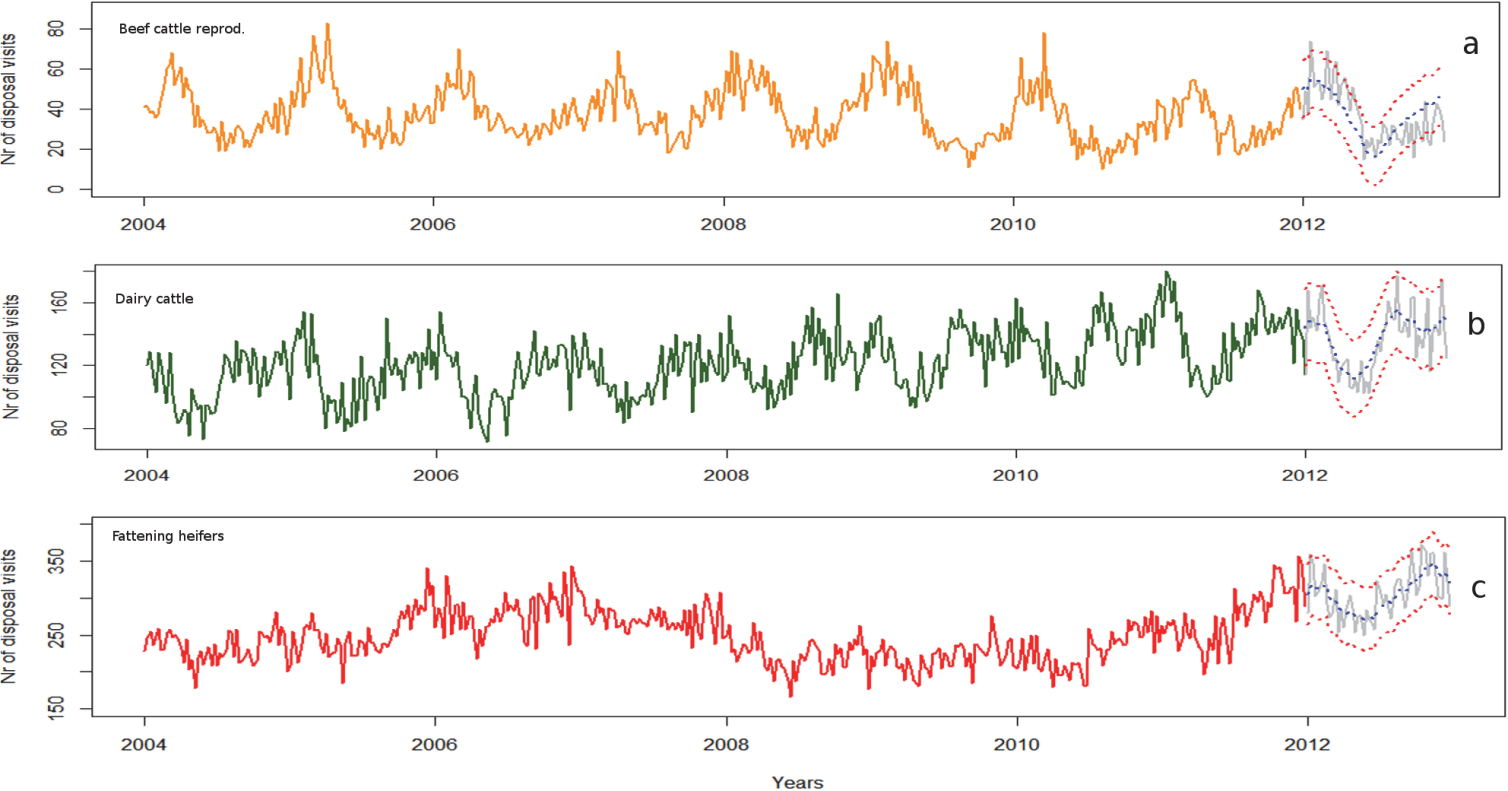
Plots of the number of carcass disposal visits for each type of production series at the regional level and forecast values using ARIMA modelling with a seasonal adjustment.

The basal patterns for disposal visits carried out between 2004 and 2011 and the forecasts estimated from these models for each of the 52 weeks in 2012, compared to the actual observations, are illustrated in Fig 3. The observed values between 2004 and 2011 are drawn as a orange line (training dataset) for beef cattle reproduction, as dark green line for dairy cattle, and as red line for fattening heifers. The grey lines represent the observations from 2012 (test datasets). The forecast for each production type is represented in each plot as a blue dotted linefor point estimates, and as red dotted lines for their 95% confidence bands.

For beef cattle reproduction, the model defined for V corresponded to an ARIMA(1,0,1) model with trigonometric seasonal component adjusted. The mean number of collections registered at the regional level per week between 2004 and 2011 was 38, within a range of 10 and 83. The variation of this series was relatively constant over time with a marked yearly seasonal component expressed in X_t_. An increase of bovine fallen stock collections was observed from October to March. The random component Y_t_ fitted to an ARIMA(1,0,1) model with a high autocorrelation between the observations t and t-1 and an inverse effect of the moving averages.

The model predicted a mean of 38 disposal visits per week within a range between 8 and 71, based on a 95% confidence band. When considering the test data from 2012, most of the real data fitted the predictions except for one abnormal peak of disposal visits detected during the first part of the year (Fig 3).

In dairy cattle farms, the disposal visits carried out by week at the regional level were fitted to an ARIMA model (1,0,1) with trigonometric seasonal and trend adjustments. This series appeared to increase over time, especially between 2007 and 2011 (see Fig 3). The increasing trend is expressed in X_t_ as the coefficient of t, and in the same equation two seasonal cycles, both half-yearly and yearly, were adjusted.

Between 2004 and 2011 the mean number of visits registered weekly in dairy cattle farms was 121 within a range of 71 to 180. Whereas, the forecasts estimated from this model for the subsequent 52 weeks (in 2012) had a mean of 146, and the estimated range based on a 95% confidence band was between 90 and 179.

### Fattening heifers

In fattening heifers the disposal visits carried out by week was fitted to an ARIMA model (0,1,1) with previous seasonal adjustment.

Between 2004 and 2011 the range of disposal visits carried out by week varied from 166 to 357 with a mean of 248. The seasonal component showed yearly variation with a small increase in the second half of the year.

In contrast to beef cattle reproduction and dairy cattle, the time series of V in heifer fattening farms presented a marked alternating trend, increasing from late 2005 to 2008, before decreasing between 2008 and 2011, and then increasing again from 2011. According to the available information collected from the field these fluctuations could not be directly associated to any unique events that occurred at the regional level. To explain these alternating patterns, further investigations comparing the time series of kg and exploring the time series at different geographical scales using HTS were necessary.

For heifer fattening farms the expected mean number of disposal visits for the 52 weeks in 2012 was 300 (within a range based on the 95% CI from 231 to 373). Fig 3C indicates the baselines for V and the forecasts estimated for the subsequent 52 weeks.

**Fig 4:**
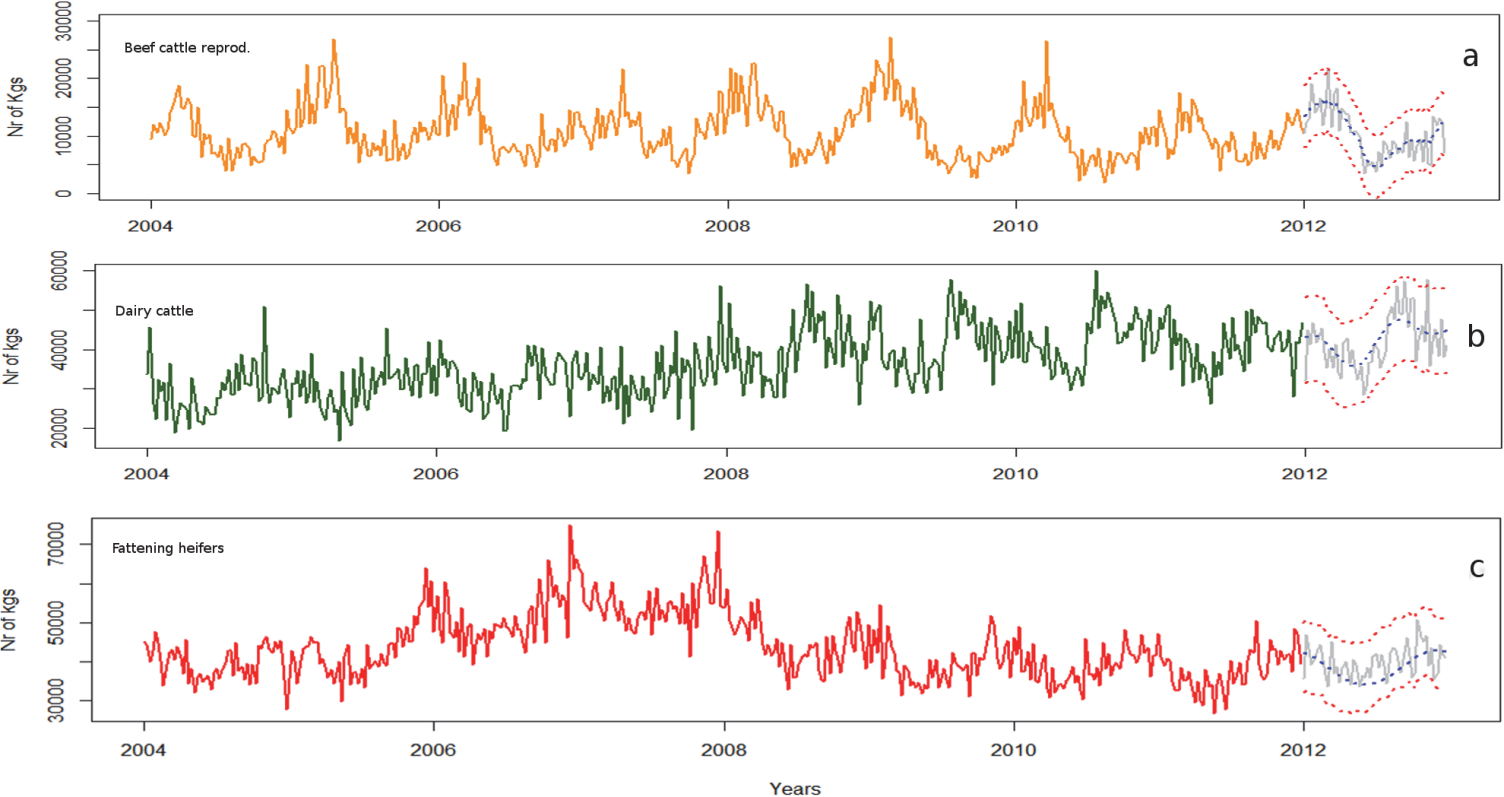
Plots of kilograms of carcasses collected by week at the regional level for each type of production based on the ARIMA model.

ARIMA modelling was also used to fit the models to kg of collected carcasses (Table 2 and Fig 4). Comparing the V and kg patterns (Figs 3 and 4), we observed that for both beef cattle reproduction and dairy cattle the trends showed similar baselines However, in the event of fattening heifers the trends of V and kg were significantly different. In this case, although the trend in kg increased between 2006 and 2008, the level of kg collected was almost constant over time with a yearly seasonal pattern from 2008.

Plots of diagnostic checks for V and each production type, using standarized residuals with their correspondent autocorrelation and partial autocorrelation, are shown in S1, S2 and S3 Figs.

### Hierarchical time series plotting

**Fig 5:**
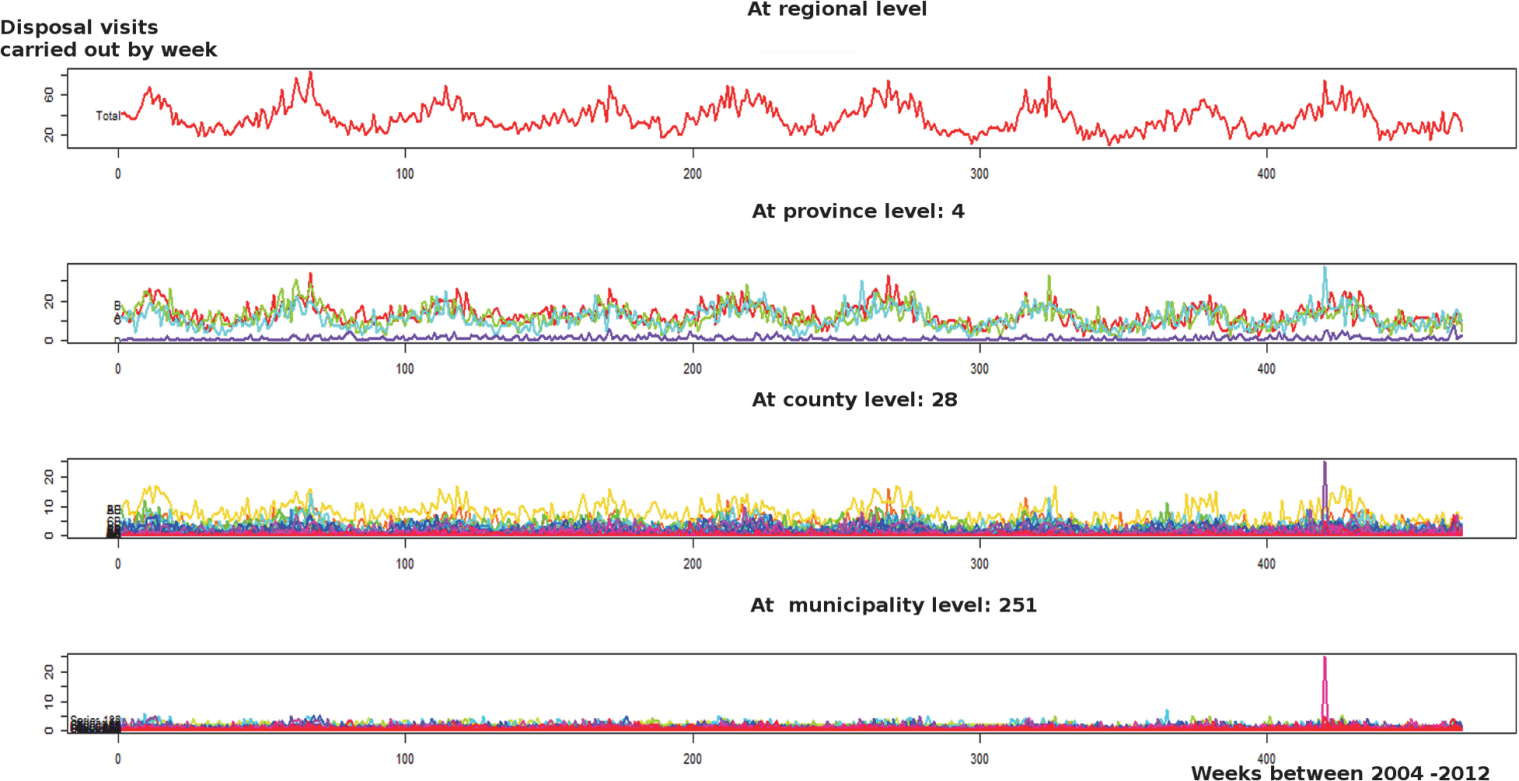
Hierarchical time series at four spatial levels based on number of carcass disposal visits, aggregated by week for beef cattle reproduction farms. A peak of mortality was evidenced at municipality scale (Week 421).

The hierarchical time series plots of V for the beef cattle reproduction series are shown in Fig 5. These plots correspond to the collections performed weekly in 757 farms, from 251 municipalities across 28 counties, located in the 4 provinces. Using this method it is apparent that there was an abnormal increase of disposal visits observed early in 2012 (see also Fig 3) which only occurred in two neighbouring municipalities (indeed it is almost indiscernible in the regional level plot).

**Fig 6:**
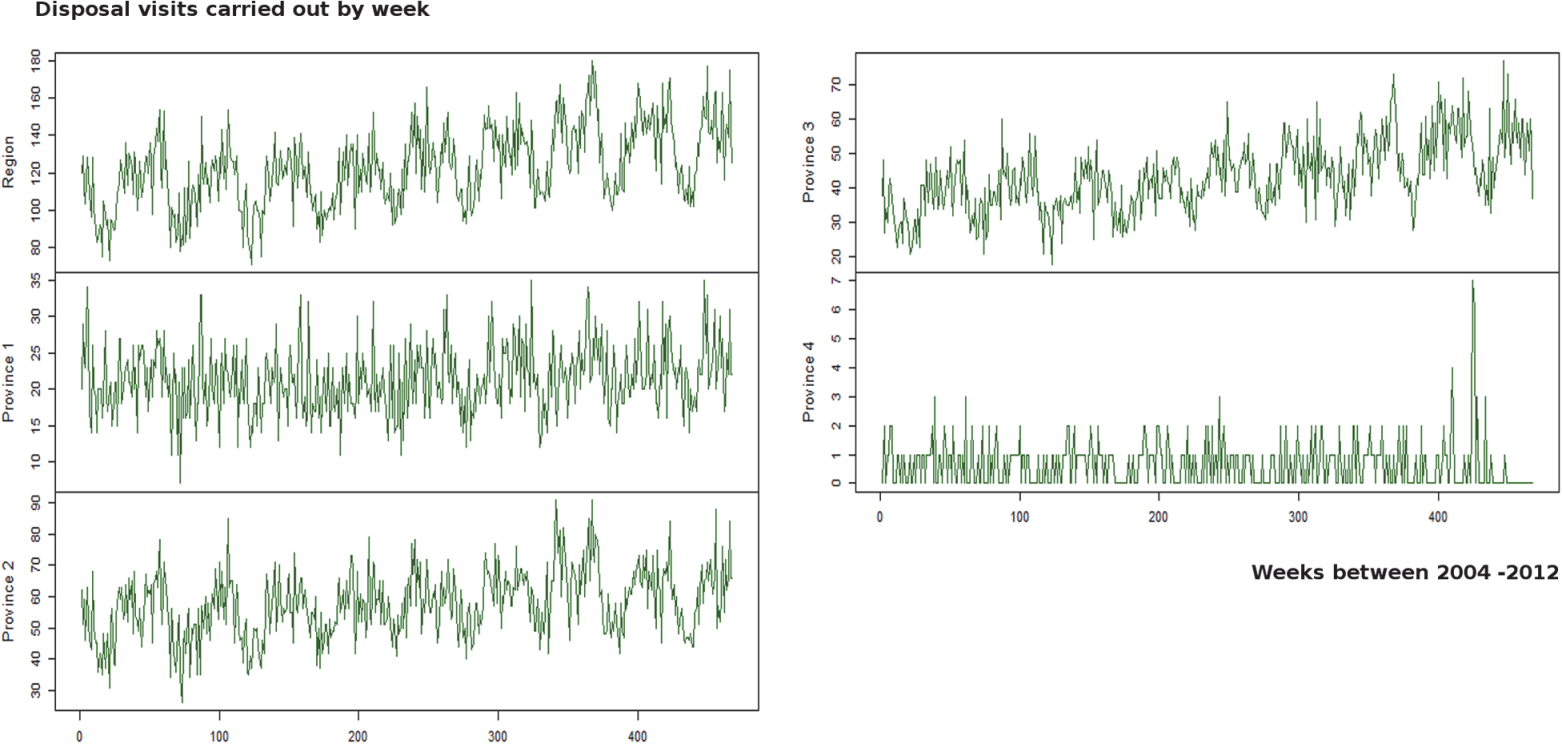
Disaggregated hierarchical time series of carcass disposal visits at regional and province level for dairy cattle farms.

The HTS for dairy cattle (Fig 6) corresponded to weekly collection visits carried out in 426 farms, from 195 municipalities across 26 counties, located in the 4 provinces. These hierarchical time series plots are by region and by each of the 4 provinces.

**Fig 7:**
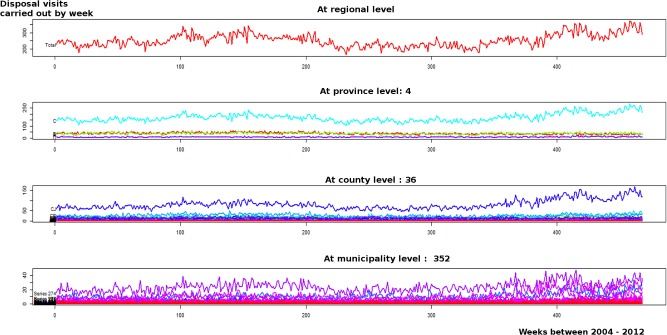
Hierarchical time series at four levels based on the number of carcass disposal visits, aggregated by week in heifer fattening farms.

Finally, for the heifer fattening farms, the HTS corresponded to collections performed in 1808 farms, across 352 municipalities in 36 counties, located in the 4 provinces (Fig 7). From these plots we can identify that the increase observed from 2011 in V was mainly associated with a single county in province 3, which has the highest concentration of heifer fattening farms.

To complement the information on the fallen stock patterns observed based on the number of visits carried out (V), the HTS plots fitted to the kg collected were also examined. Comparing V and kg without taking into account their respective measurement scales, we observed that the outcomes for both beef cattle reproduction and dairy cattle farms showed similar patterns. In contrast, for heifer fattening farms the patterns associated with V and kg collected, differed markedly. In particular, in assessing the HTS plots for the number of kg of carcass collected from heifer fattening farms, we observed that the trends of kg collected did not increase between 2010 and 2012 (shown as supporting information in S4 Fig).

### Interpretations from the time series analyses

The seasonal patterns observed for each production type could be mostly explained by reproductive management factors. In the case of beef cattle reproduction, the seasonal pattern associated with fallen stock coincided with the period of calving and confinement [19–20]. Whereas, in dairy and heifer fattening farms, the seasonal pattern was not only associated with the reproductive management systems, but also with the periods of thermal stress in summer or respiratory disorders aggravated by cold climatic conditions [21–24].

In addition, based on the interviews conducted, other plausible explanations for specific patterns were uncovered. The veterinary practitioners interviewed explained that the increasing trends observed in both dairy from 2007, and heifers between 2006 and 2008, were most likely associated with rising production costs and/or the implementation of an EU-wide ban on antibiotics, previously used as growth promoters in animal feed [11].

In contrast, the increasing trend of disposal visits to heifer fattening farms observed from 2010, mostly related to a single county, was associated with changes in the logistics of carcass collection in that area and not with any general increase of cattle mortality (a fact that could also be surmised from the time series that were fitted to the weight of carcasses collected).

## Discussion

Previous studies have proposed the potential of mortality data to monitor the health state of animal populations, the evolution of some types of epidemic, the early detection of unexpected events and an assessment of the impact of various abnormal events that have occurred in the past [4–7]. However, due to their unspecific nature and the large variability in the animal populations from which these data originate, their use as an approach for detecting early health related events constitutes a challenging task that requires a multidisciplinary and ordered approach.

The development of an appropriate early detection system would consist of a number of parts which should be constantly checked: identification of data sources, data collection, data warehousing, exploratory data analysis to ensure representativeness, cluster analysis to determine groups to be studied, time series plots, identification of algorithms, testing of various forecasting strategies to select the most appropriate models, and the validation of these forecasts [25]. This study has proposed a combination of classical and novel methods and their strengths and weaknesses observed in the context of this study are discussed to assist those considering their use in similar contexts.

The first constraint for building any system in the current context was the impossibility of obtaining the exact number of dead animals at the farm level. However, proxy measurements, such as the number of collections carried out and the number of kilograms of carcasses collected, were available. These data were automatically recorded in a dataset spanning a period of nine years and potential information on mortality patterns could be extracted. In the context of cattle farms, most of the times that a carcass disposal visit occurred corresponded to the collection of a unique bovine carcass (since the farmer called the carcass collection service as soon as he/she detected a deceased bovine on the farm). Because of this, we assumed that the number of disposal visits could serve as an indirect indicator of the animals that died at the farm level. To complement the results from these observations, the weight of carcasses collected was also analysed in parallel.

For interpreting the patterns observed, we assumed that an increasing trend at the same time for both outcomes (V and kg) would tend to be associated with an increase in animal mortality; however, when the trends differed between outcomes other causes, such as changes related to the logistics of collection, might be also postulated. It should be noted that initially the adjusted trends of the proposed models were fitted as linear patterns. This approach aimed to reduce the number of parameters required and simplify plausible explanations. Nevertheless, more complex trend patterns could be adjusted for by using segmented linear trends or other polynomials of a higher order.

Using the ARIMA modelling, our work aimed not only to identify and compare the historical baselines of bovine fallen stock in the main types of productions of cattle at the regional level, but also to characterise their seasonality and trend in a comprehensive manner. Moreover, when the time series presented regular patterns without including zeros in the observations, this modelling approach could predict events and provided evidence of the existence of irregularities. These results were in accordance with recent studies in similar contexts [26–29]. However, it should be noted that if the time series contains many zeros or very low values, ARIMA approach is likely to be less well suited and alternative modelling options should be explored. In this case, one of these options could be the integer-valued autoregressive models that are suitable for fitting count time series to Poisson distributed patterns [30].

Furthermore, the evidence of heterogeneous mortality patterns among the three production types and within provinces, counties, and municipalities also emphasised the importance of studying these patterns at appropriate geographical scales and pointed to the importance of defining consistent clusters to determine robust algorithms for the time series observed. In this sense, the use of hierarchical time series was found to be a powerful method for comparing the patterns associated with different subpopulations over time, for assessing the extent of irregularities observed and for identifying abnormalities in limited animal subpopulations. Moreover, provided that the model structure at the regional level has been confirmed, subpopulations at lower levels that presented similar patterns could be modelled using the same model structure. Although this approach had been used in other fields [18], as far as we know this was the first time that this methodology had been applied to veterinary syndromic surveillance.

However, if this system had to be used as an early detection method of diseases, we had to consider that variations in the levels of fallen stock might not be influenced only by disease incursion, but also by other factors (such as climatic variation, limited availability of feeding in pastures, production costs and benefits, trade, implementation of new legislative rules, specific conditions at the farm level and available logistics for disposal collections) [4–6]. In this sense, the incorporation of additional data as covariates (such as continuous updates of census data, animal age, breed, production or environmental parameters) would be useful to clarify the causal mechanisms associated with mortality peaks, to improve the grouping strategies, and thus decrease the amount of unexplained error.

This work corroborates that when unspecific data is used as a health indicator, the interpretation of resulting models requires cautious assessment. In this study, gathering information from the field about the methods of data collection and the analysis of other possible indicators, proved critical for explaining more accurately the observed patterns. This was most clearly exemplified in the case of heifer fattening farm data.

In conclusion, despite the unspecific nature of fallen stock data, the utility of such data in conjunction with other parameters has been demonstrated to be useful as an indicator of cattle health. The analyses conducted in this study were based on unspecific data consistently registered from a reasonable coverage of the bovine population at the regional level. These data provide valuable information for epidemiological surveillance of animal population health. Moreover, the classical and novel approaches presented in this study may help not only to design more efficient systems of syndromic surveillance, but also to assess the impact of health intervention measures applied or changes that occur over extended time periods. However, to implement such system as components of syndromic surveillance, further research must be carried out in order to identify specific causes of mortality peaks, remove abnormal events from basal patterns, and to re-test and validate the novel algorithms.

## Supporting Information

S1 Fig Plots of the diagnostic checks of residuals from the ARIMA modelling from the number of carcass disposal visits for beef cattle reproduction farms. (TIF)

S2 Fig Plots of the diagnostic checks of residuals from the ARIMA modelling from the number of carcass disposal visits for dairy cattle farms. (TIF)

S3 Fig Plots of the diagnostic checks of residuals from the ARIMA from the kilograms of carcass collected for heifer fattening farms. (TIF)

S4 Fig Plots of hierarchical time series at the four spatial aggregation levels fitted to the data of kilograms of bovine carcasses collected weekly in heifer fattening farms. (TIF)
