# Supplementary figures and images for: Exploring the Surveillance Potential of Mortality Data: Nine Years of Bovine Fallen Stock Data Collected in Catalonia (Spain)
Source: PLoS One. 2015 Apr 15;10(4):e0122547. doi: 10.1371/journal.pone.0122547 (PMC4398401; doi:10.1371/journal.pone.0122547)

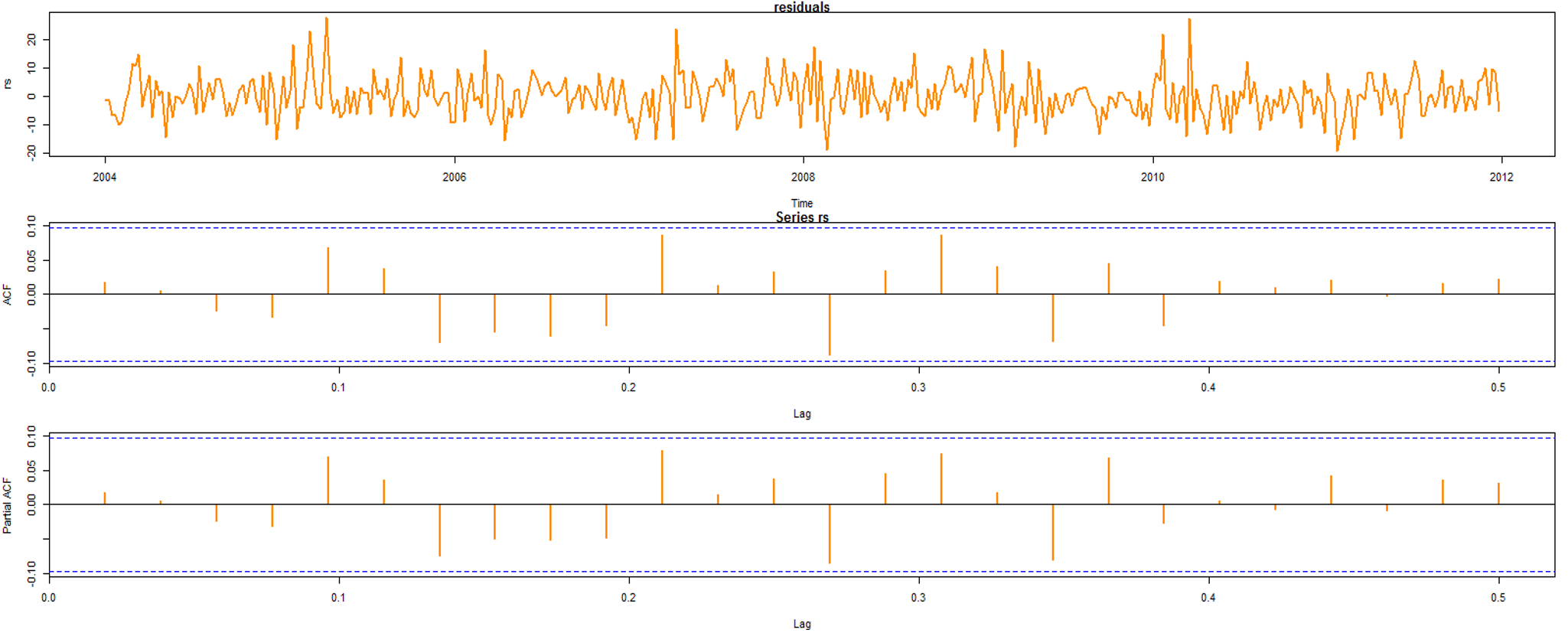

Supplement: S1 Fig — (TIF) [file pone.0122547.s001.tif]

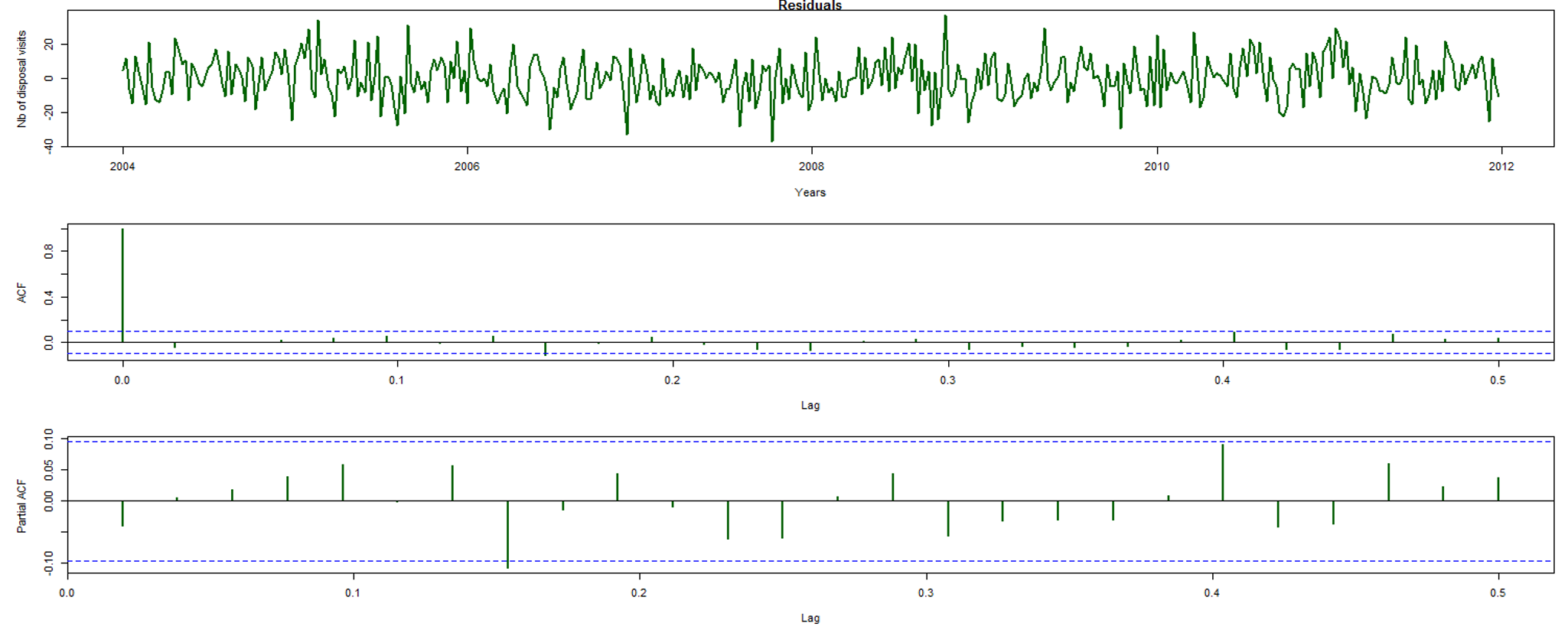

Supplement: S2 Fig — (TIF) [file pone.0122547.s002.tif]

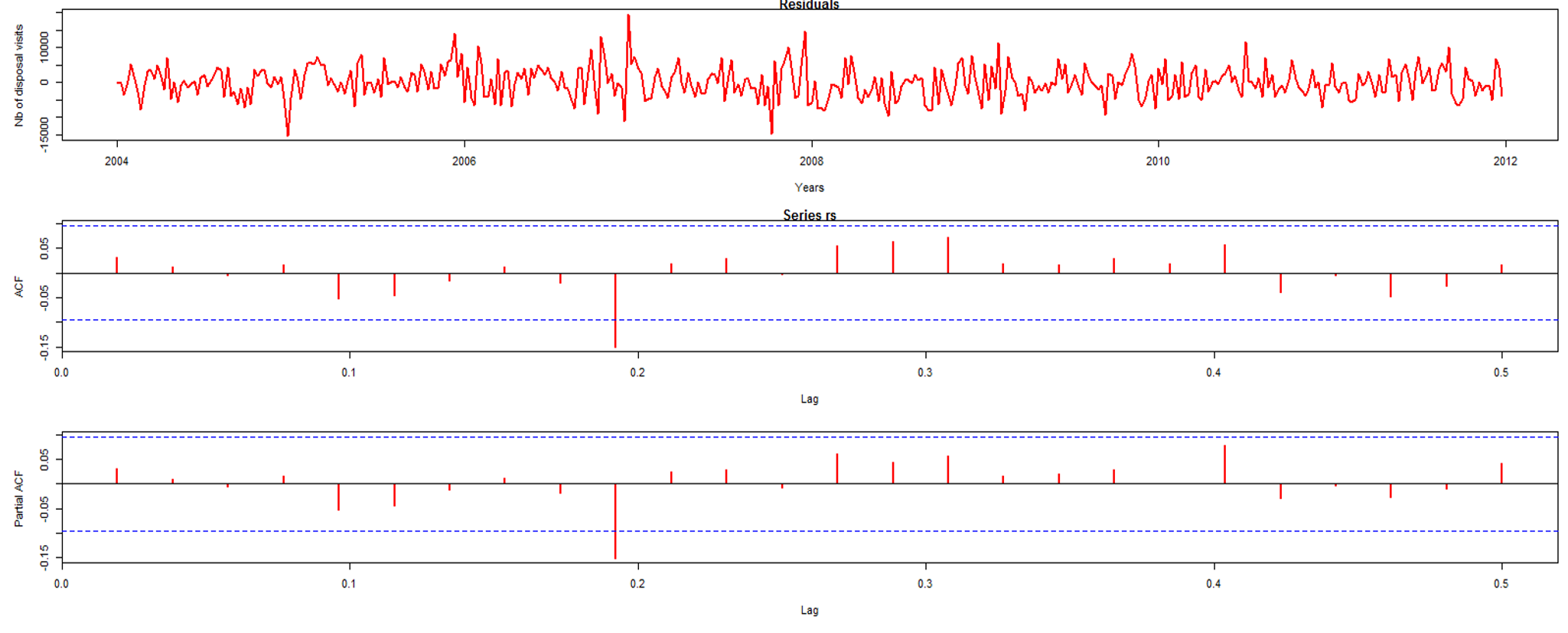

Supplement: S3 Fig — (TIF) [file pone.0122547.s003.tif]

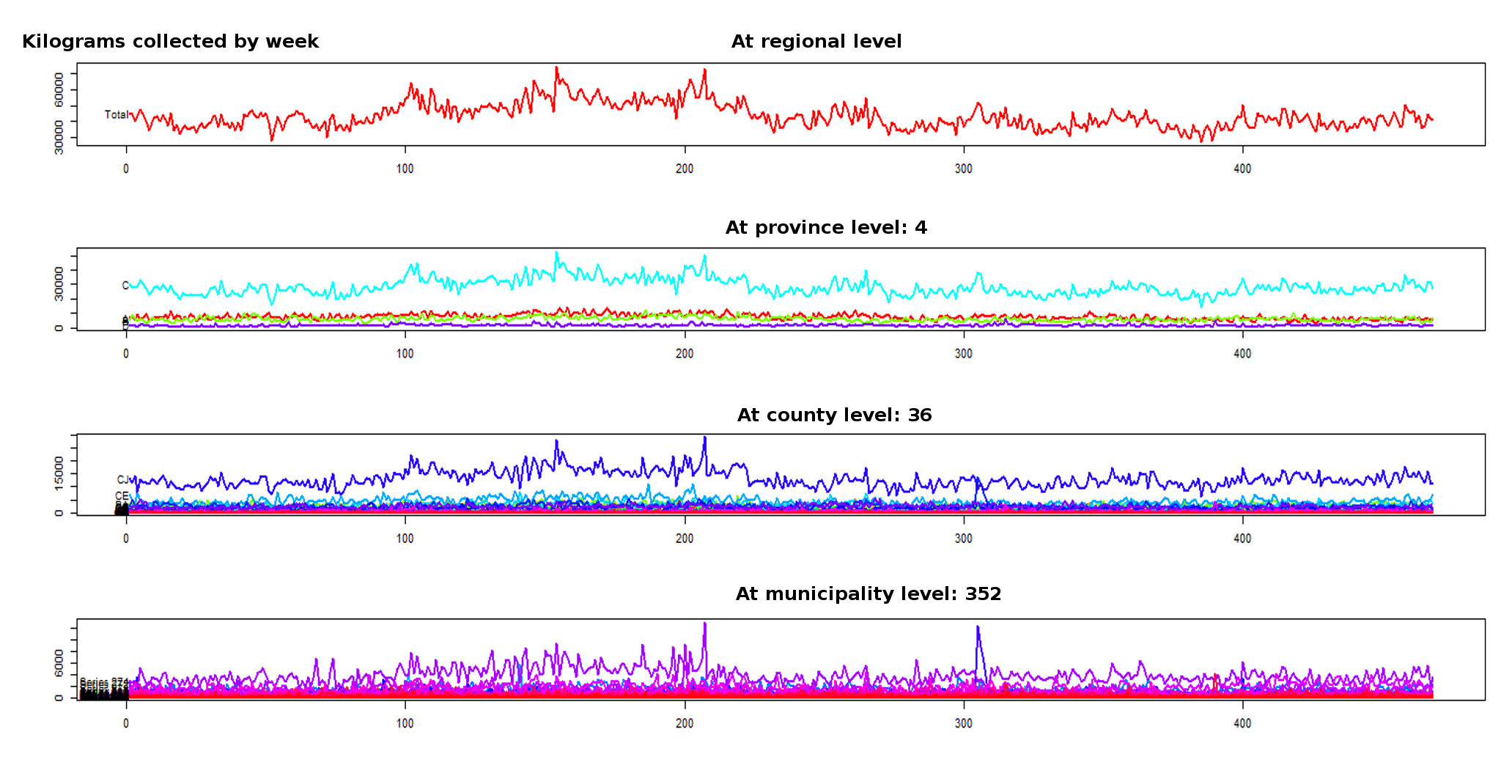

Supplement: S4 Fig — (TIF) [file pone.0122547.s004.tif]
